# Development of Composite Indices to Measure the Adoption of Pro-Environmental Behaviours across Canadian Provinces

**DOI:** 10.1371/journal.pone.0101569

**Published:** 2014-07-11

**Authors:** Magalie Canuel, Belkacem Abdous, Diane Bélanger, Pierre Gosselin

**Affiliations:** 1 Institut national de santé publique du Québec (INSPQ), Québec City, Canada; 2 Centre de recherche du Centre hospitalier universitaire de Québec, Québec City, Canada; 3 Département de médecine sociale et préventive de l′Université Laval, Québec City, Canada; 4 Institut national de la recherche scientifique, Centre Eau Terre Environnement, Québec City, Canada; University of Waikato (National Institute of Water and Atmospheric Research), New Zealand

## Abstract

**Objective:**

The adoption of pro-environmental behaviours reduces anthropogenic environmental impacts and subsequent human health effects. This study developed composite indices measuring adoption of pro-environmental behaviours at the household level in Canada.

**Methods:**

The 2007 Households and the Environment Survey conducted by Statistics Canada collected data on Canadian environmental behaviours at households' level. A subset of 55 retained questions from this survey was analyzed by Multiple Correspondence Analysis (MCA) to develop the index. Weights attributed by MCA were used to compute scores for each Canadian province as well as for socio-demographic strata. Scores were classified into four categories reflecting different levels of adoption of pro-environmental behaviours.

**Results:**

Two indices were finally created: one based on 23 questions related to behaviours done inside the dwelling and a second based on 16 questions measuring behaviours done outside of the dwelling. British Columbia, Quebec, Prince-Edward-Island and Nova-Scotia appeared in one of the two top categories of adoption of pro-environmental behaviours for both indices. Alberta, Saskatchewan, Manitoba and Newfoundland-and-Labrador were classified in one of the two last categories of pro-environmental behaviours adoption for both indices. Households with a higher income, educational attainment, or greater number of persons adopted more indoor pro-environmental behaviours, while on the outdoor index, they adopted fewer such behaviours. Households with low-income fared better on the adoption of outdoors pro-environmental behaviours.

**Conclusion:**

MCA was successfully applied in creating Indoor and Outdoor composite Indices of pro-environmental behaviours. The Indices cover a good range of environmental themes and the analysis could be applied to similar surveys worldwide (as baseline weights) enabling temporal trend comparison for recurring themes. Much more than voluntary measures, the study shows that existing regulations, dwelling type, households composition and income as well as climate are the major factors determining pro-environmental behaviours.

## Introduction

A significant source of pollution to our natural environment comes from domestic activities and behaviours. For example household-generated waste in Canada accounts for around a third of total waste and household energy use and municipal water consumption for 17% and 57%, respectively [1]-[3]. Also, 46% of greenhouse gas emissions (GHG), which contribute to climate change, come from direct and indirect household emissions [4]. The impacts of such household pollution can be important.

Municipal waste can impact the environment in various ways including soil and water contamination from leachate in landfills disposal and the production of greenhouse gas emissions (GHG) and air pollution, either from landfills or the incineration process. When solid waste are recycled or composted instead of being landfilled or incinerated, the demand for energy and new-resources can be reduced significantly [3].

The production of energy can impact the environment in various ways, depending on the technology. In Canada, energy production and consumption accounts for around 80% of all GHG emission [5]. A household can reduce its emission of GHG by reducing electric power use. For instance high energy efficiency electronic devices or cleaner energy sources will generate less pollution and GHG.

Water shortages are happening worldwide and one way to limit their occurrence is through water conservation behaviours. In most homes, more than 60% of water use comes from toilet flushing, showers and baths, making water-saving devices like low-flow shower head an efficient way of reducing water consumption. In summer, water use can increase by 50% for yard activities such as watering the lawn. There are behaviours that households can implement to decrease their water consumption in summer time like using sprinklers with a timer or adopting the use of a rain barrel [6].

It thus becomes clear that addressing sustainability concerns has to take into account not only industry or agriculture, but also household behaviours, their impacts on ecosystems and ultimately on human health. Monitoring trends of household behaviours can inform policy and research agendas on the development of incentives or other mechanisms such as information campaigns to reduce domestic pollution and facilitate adaptative measures to minimize related health risks. The adoption of several pro-environmental behaviours, i.e. actions that contribute to the preservation of the environment, should be encouraged to significantly reduce the anthropic impact on the environment.

**Figure 1:**
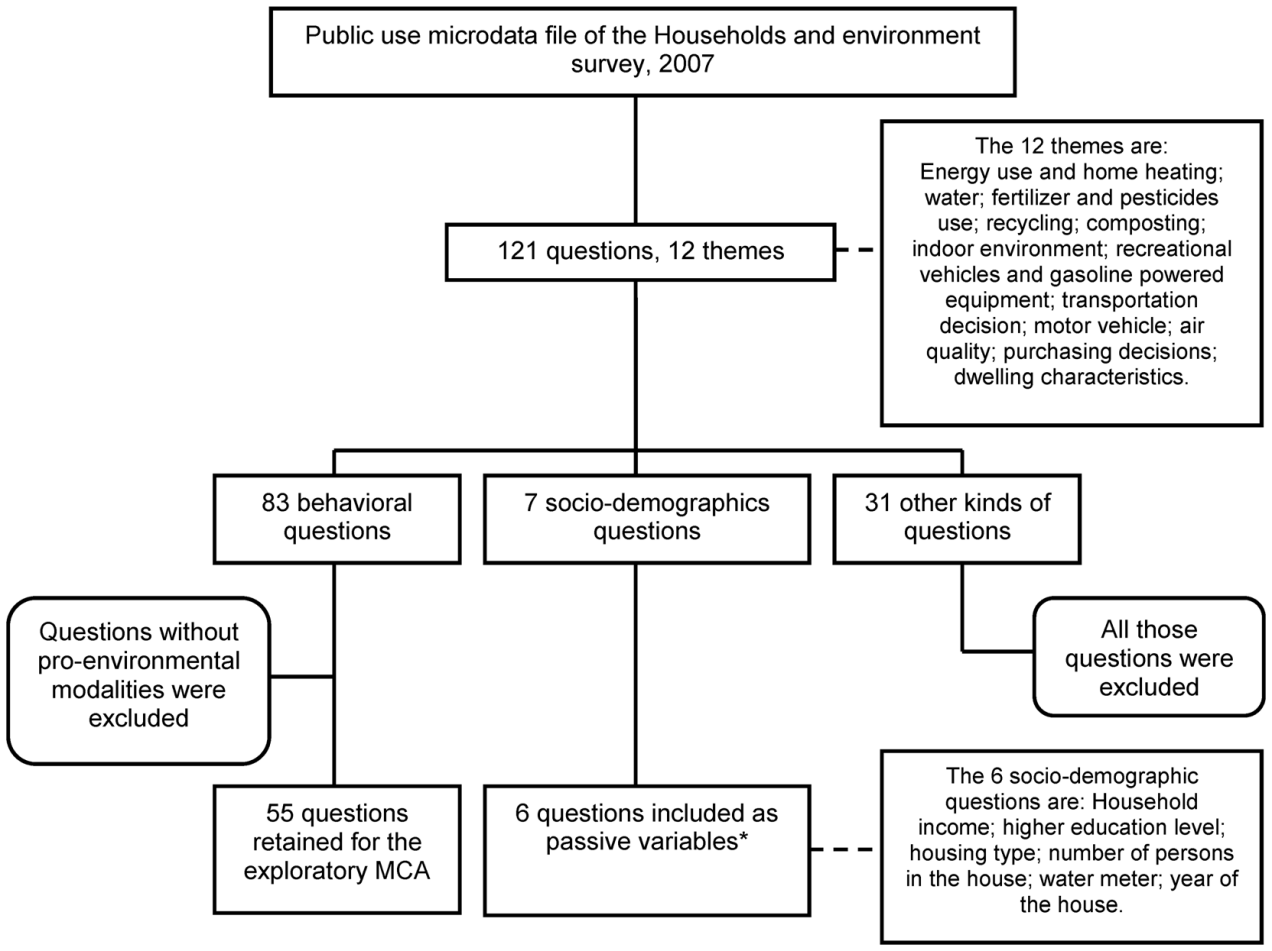
Number and type of questions selected to develop the composite index. **Legend:** *The composite index was also created for the 7th socio-demographic variable, the census metropolitan area (n = 33), but is not presented in this article.

In Canada, the Households and the Environment Survey (HES) was designed to measure household behaviours with respect to the environment. The HES is a periodic survey conducted by Statistics Canada, the federal government statistical agency, and administered across Canadian provinces. The survey covers 12 broad themes including energy use and heating, water use, transportation decisions, motor vehicle use, recycling and composting (Figure 1) [7]. While this survey provides various estimations of up to 83 Canadian practices (Figure 1) as well as some information on their socio-demographic characteristics, survey reports are limited to analyses of simple cross-tabulation frequencies for some of the 83 separate behaviours [7]-[13].

It is difficult to follow up on such a wide array of relevant behaviours and their trends over time, unless they are summarized in some way. A composite index is a tool which can be useful to that purpose as it incorporates several aspects of an issue and allow for monitoring across several themes simultaneously, thus facilitating the measurement of trends [14]. While other environmental indices exist, such as the environmental sustainability index [15], to our knowledge no index currently exists to reflect trends of pro-environmental behaviours at the household level in Canada.

This study thus sets out to develop a composite index that summarizes pro-environmental behaviours at the household level across Canadian provinces based on the HES (2007) given the periodicity and geographical coverage of the survey. Pro-environmental behaviours are defined as actions that contribute to the preservation of the environment and can have a positive impact on the health of the population. This study will serve as baseline of the trend of the composite index over time, given the periodicity and geographical coverage of the survey.

## Materials and Methods

### Ethics statement

This research did not require the approval of an ethics review board as we used an existing and anonymized database made available to universities by Statistics Canada. Statistics Canada obtained consent previous to survey administration. No new data was collected for this study.

### Survey

The Households and the Environment Survey (HES) is conducted by Statistics Canada. It was designed to address the needs of the Canadian Environmental Sustainability Indicators project. The project reports on air quality, water quality and greenhouse gas emissions in Canada using indicators to identify areas of importance to Canadians and monitor progress [16].

The survey aimed Canadian households with at least one person aged 18 year or older. The HES covers all 10 of the provinces and excludes the 3 northern territories, Indian reserves and members of the Canadian Armed Forces. The survey was first conducted in 1991 and since 2005 has been carried out biennially. In the present study, the 2007 HES database was used in its Public Use Microdata Files format (PUMF) [16]. As a sub-sample of the dwellings that were part of the Canadian community health survey (CCHS), the sampling allocation for the HES followed that of the CCHS closely. The CCHS used a multistage stratified cluster design in which the dwelling is the final sampling unit. Three sampling frames were used to select the sample of households: 50% of the sample came from an area frame, 49% came from a list frame of telephone numbers and 1% came from a Random Digit Dialing sampling frame [16].

From the 40 584 households selected in the 2007 CCHS, a sub-sample of 29 957 households were selected for the HES. Of those, 21 690 households responded to the survey resulting in an overall response rate of 72%. The survey is representative of 12 932 350 households, corresponding to 97% of all Canadian households [16]. The questionnaire was administered to the 21 690 households by telephone interview spread over a 6-month period, from October 2007 to February 2008.

### Questionnaire

The person with the best knowledge of environmental household practices was asked to respond on behalf of the household. The main questionnaire covered 12 themes and included 121 questions (figure 1) [16]. Among the questions, 83 measured behaviours and 7 measured socio-demographic characteristics. The other 31 questions covered knowledge, reasons for not adopting the behaviour, or served to specify some characteristics (e.g. of a good) or to filter for the next question.

### Database

The PUMF was used for the analysis and unlike the master file, applies privacy measures to protect personal information [16]. In the PUMF, data were mostly coded as categorical variables. Three different labels (don't know, not stated, and refusal) were used to classify households who did not participate despite eligibility or to protect the anonymity of the household. A ‘valid skip’ label was used when the provision of a response was not appropriate. For example, a household who answered ‘no’ to the question for car ownership was allocated a ‘valid skip’ label for subsequent questions on the characteristics of the car.

### Sampling weights

Sampling weights were applied to ensure that any derived composite index is representative of the study population. They were used when proportions and averages were estimated and to weight the relative frequencies of the Burt matrix in the MCA (see Statistical analysis below).

### Variables selection

This study focuses on everyday pro-environmental behaviours, defined as actions that contribute to the preservation of the environment and can have a positive impact on the health of the population. For example, air pollutants can be reduced when households adopt behaviours that decrease their energy consumption such as the use of energy-efficient appliances or when they use more sustainable transport options such as public or active transport.

Based on the above definition, a panel of four environmental health experts applied progressive development consensus after iterations, based on a nominal group technique [17] to evaluate HES variables for exclusion. These were either: variables not measuring a behaviour or questions with no clearly pro-environmental response option. Socio-demographic variables were kept as passive variables with zero mass and no influence on the analysis. They support and complement the interpretation of the map representation of the active variables [18].

### Statistical analysis

Given that the data was mostly categorical, the indices in this paper were developed by multiple correspondence analysis (MCA) [18]. Several authors have used Multiple Correspondence Analysis (MCA) as a weighting method for the construction of a composite index [19]–[23]. MCA is a data reduction procedure for categorical variables (nominal or ordinal) as much as Principal Components Analysis is for quantitative variables [18]. It enables the exploration of associations within a set of variables by transforming the whole data set into dummy variables to form an indicator matrix or upon construction of a matrix from all two-way cross-tabulations among the variables (Burt matrix). This transformed data is treated as a cloud in a space equipped with the classical Chi-square distance. This distance is used in the assessment of homogeneity and variance (inertia) of rows or columns of the indicator or Burt matrix. The most crucial step of MCA is its use of singular value decomposition and weighted least squares techniques to find low-dimensional best fitting subspaces with minimal inertia and information loss [18].

MCA was conducted using the ‘ca’ package of the R statistical software [24]. First, the HES database was converted to a Burt matrix taking into consideration the sampling weights. A Burt matrix is a square symmetric categories-by-categories matrix formed from all two-way contingency tables of pairs of variables [18].

Then, an exploratory MCA was performed to project data onto maps where potential outliers were identified and excluded from subsequent analyses. MCA was then applied again to determine the most relevant factorial axes that would serve to build the composite index. There are no universal rules for the determination of the number of dimensions to retain in MCA. However, since the first factorial axis captures the most important part of the total inertia, it plays a central role in the computation of a composite index.

As recommended by Asselin [23], we sought questions having the property of First Axis Ordering Consistency (FAOC). To this end, we projected all the questions on the first axis and tried to identify those having an ordinal structure consistent with respect to this axis, i.e. all questions with pro-environmental responses improving from left to right (or conversely).

The computation of the index score was performed as follows: first, the score of any household was obtained by taking the average of its category-weights generated by the MCA. Then for each province we took the average over all household scores as the value of its composite index. The sampling weight was used in this final step. Coordinates were missing for excluded responses.

The 10 average provincial scores were grouped into categories reflecting different levels of adoption of pro-environmental behaviours. First we applied a cluster analysis and then we used a dendrogram plot using SAS version 9.2 (SAS Institute, Cary, NC) to determine such groups. The categories limits generated for the provincial index were used as reference categories for indices on other socio-demographic variables.

Finally, others indices based on various socio-demographic variables were constructed (Figure 1). Household scores were calculated by taking the average of its category-weights generated by the MCA. Then the index score of the socio-demographic category (e.g. household with annual income less than $40,000) is set as the average of the corresponding household scores.

## Results

### Multiple Correspondence Analysis

**Figure 2:**
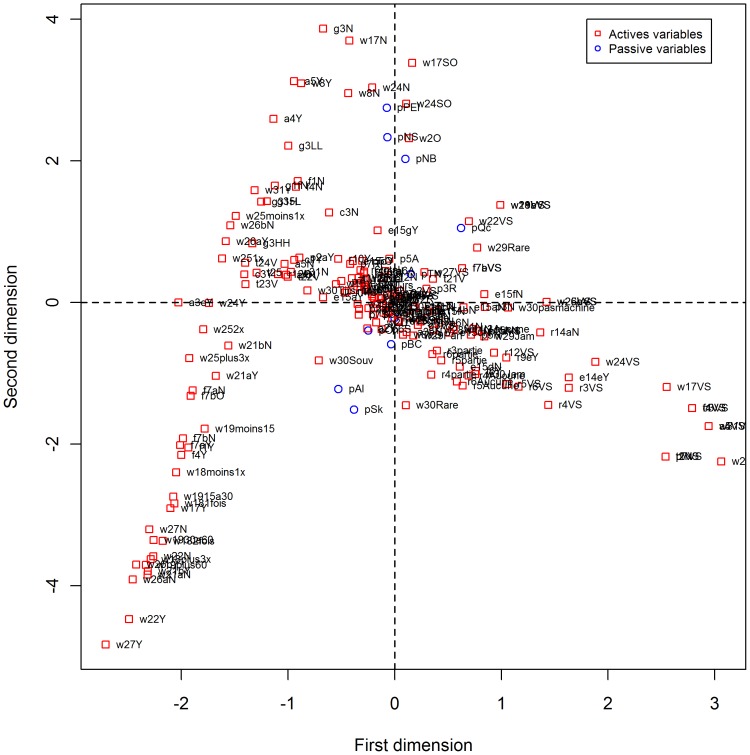
Map representation of the MCA results on the 55 questions without extreme responses.

Of the 121 questions in the survey, 55 were kept by the Expert Panel for use in the MCA. These represented 285 response possibilities. On the MCA map projection there was a clear opposition between the missing data (don't know, refusal, not stated) located far from the map center and the other responses which gathered close to the center (Figure S1). Excluding the missing data rebalanced the model (179 remaining responses) (Figure 2). However, since pro-environmental behaviours were spread over both sides of the first axis, we failed to find any meaning to this first dimension.

We then screened the projected responses to identify questions following an ordinal structure, (i.e all pro-environmental responses of a question have negative coordinates on the first factorial axis (or conversely)). Twenty-three such questions with pro-environmental responses deteriorating from left to right on the first axis (group A), and 16 questions with opposite ordinal structure (group B) were identified. The remaining 16 (of 55) questions were excluded from the analysis because their responses were not sufficiently discriminating (i.e. the pro- and anti-environmental responses were on the same side of the axis or they were grouped close together on the map). As well the majority of these questions (10/16) had at least two responses with a contribution of zero to the first axis (Table S1).

**Figure 3:**
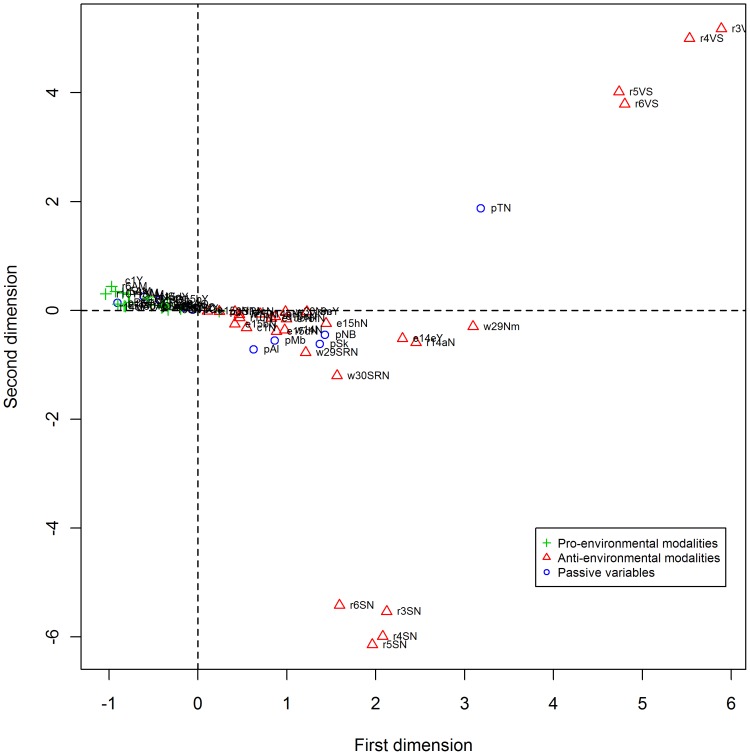
Map representation of the MCA results on the 23 questions of the group A (Indoor).

These exploration steps led us to consider two separate composite indices. Group A included 96 responses but after excluding missing data, 52 responses were used in the MCA. The majority of excluded responses had frequencies lower than 2.0% and two responses had frequencies of 4.6% and 4.7%. After exclusion of missing data, some responses still looked like extreme values on the map (Figure 3). They were kept in the analysis as they are 2 of the 3 responses for all questions concerning recycling. Excluding these responses would have resulted in the exclusion of all recycling questions. Responses used in this analysis had a frequency of 7.5% or higher, except for two responses with frequencies of 2.5% and 3.5% (responses on recycling).

For group A, the first dimension explained 32.6% of the inertia while the second explained 16.1% (Table 1). Given that the first factorial axis plays a central role in the construction of this composite index, only the first dimension was selected to construct the index. This group respects the FAOC as pro-environmental responses are located on the left of the first axis as opposed to others responses deteriorating to the right (Figure 3). Also, we noted that the retained questions were associated with five themes of the survey: energy use and home heating, water, recycling, composting and, purchasing decisions. All 23 questions assessed behaviours practiced inside the dwelling and thus the first axis measures these behaviours. Twelve of 15 responses contributing the most to the first factorial axis concerned recycling (Table S2).

**Table 1:** Explained inertia by each dimension for group A: Indoor Index, 2007.

Dimension	Inertia	Inertia (%)	cumulative Inertia (%)	*scree plot*
1	0,0270	32,6	32,6	*************************
2	0,0133	16,1	48,8	************
3	0,0078	9,4	58,2	*******
4	0,0032	3,8	62,0	***
5	0,0030	3,6	65,6	***
6	0,0026	3,2	68,8	**
7	0,0024	2,9	71,7	**
8	0,0021	2,5	74,2	**
9	0,0019	2,3	76,5	**
10	0,0018	2,1	78,7	**
11	0,0017	2,0	80,7	**
12	0,0016	1,9	82,6	*
13	0,0015	1,8	84,4	*
14	0,0014	1,7	86,1	*
15	0,0014	1,7	87,7	*
16	0,0013	1,6	89,3	*
17	0,0012	1,5	90,8	*
18	0,0012	1,4	92,2	*
19	0,0011	1,3	93,5	*
20	0,0011	1,3	94,8	*
21	0,0010	1,2	96,1	*
				
52	0	0,0	100,0	

**Figure 4:**
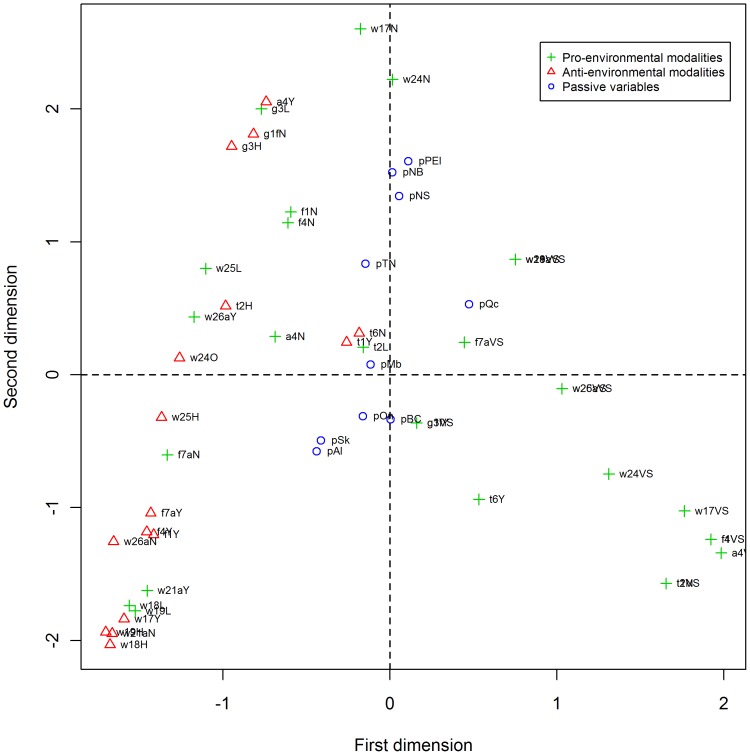
Map representation of the MCA results on the 16 questions of the group B (Outdoor).

The second group of 16 questions (group B) consisted of 86 responses, 41 of which were missing values. The 45 remaining responses used for the MCA had frequencies of 7.0% or higher while excluded responses had frequencies lower than 3.5%. For group B, the first dimension explained 62.1% of the inertia while the second explained only 12.4% (Table 2). Again, the first dimension was selected for the construction of the index and pro-environmental responses were located on the right of the first axis with other responses deteriorating from right to left (Figure 4). The 16 questions cover five themes of the survey: water, fertilizer and pesticide use, recreational vehicles and gasoline powered equipment, transport decisions and air quality, all behaviours being practiced outdoors. Of note, 9 of the 15 responses contributing the most to the first factorial axis concern households with no lawn or garden (i.e. the application of fertilizers or pesticides, yard waste and watering of the lawn or the garden) (Table S3).

**Table 2:** Explained inertia by each dimension for group B: Outdoor Index, 2007.

Dimension	Inertia	Inertia (%)	Cumulative inertia (%)	*scree plot*
1	0,2173	62,1	62,1	*************************
2	0,0434	12,4	74,5	*****
3	0,0162	4,6	79,1	**
4	0,0129	3,7	82,8	*
5	0,0111	3,2	86,0	*
6	0,0094	2,7	88,7	*
7	0,0066	1,9	90,6	*
8	0,0061	1,7	92,3	*
9	0,0041	1,2	93,5	
				
45	0	0,0	100,0	

Because two distinct behavioural categories resulted from the MCA, two composite indices were created instead of one. The first index (group A) is named the ‘Indoor Index’ and the second one (group B) the ‘Outdoor Index’. Questions included for each index are presented in supporting information, Table S4 and Table S5.

### Composite indices by province

The map representations of the final coordinates generated by the MCA are shown in Figure 3 and Figure 4. Coordinates and other results of the MCA are available in supporting information, Table S2 and Table S3. The coordinates of the first dimension were used to construct each of the two composite indices. Coordinates are missing for responses that have been excluded. Only 0.9% and 0.4% of coordinates are missing for the indoor and outdoor indices, respectively.

For the Indoor Index, the households belonging to a province with negative coordinates tend to adopt more pro-environmental behaviours than those of a province with positive coordinates. In contrast, for the Outdoor Index provinces with positive coordinates adopt more outdoor pro-environmental behaviours than those with negative coordinates.

**Figure 5:**
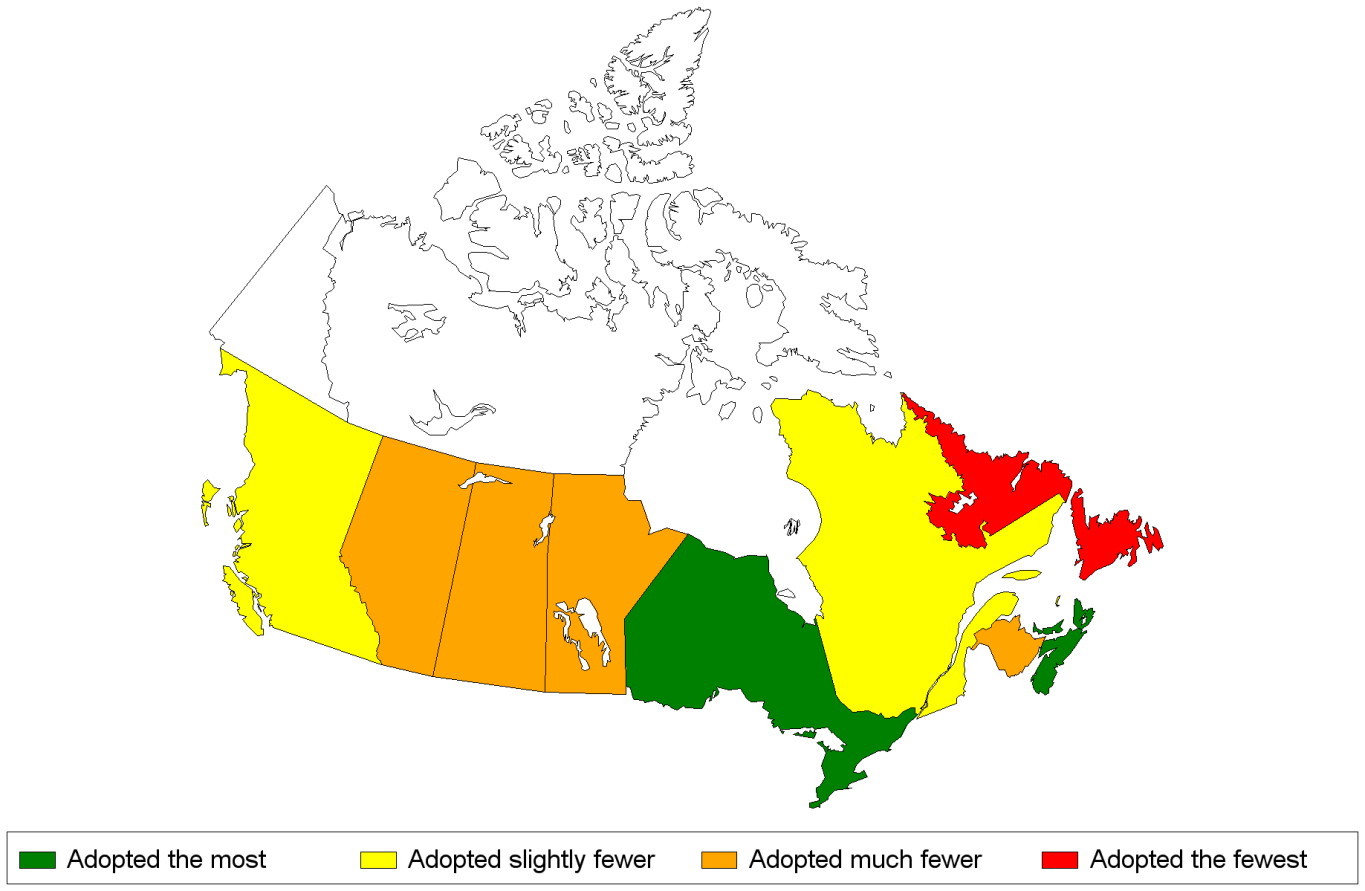
Provinces' classification according to the four categories of pro-environmental behaviours, Indoor Index, 2007. **Legend**: from left to right – British-Columbia, Alberta, Saskatchewan, Manitoba, Ontario, Quebec, New-Brunswick, Nova-Scotia. Prince-Edward-Island is North of the two latter provinces and Newfoundland-and-Labrador is located North-East of Quebec.

**Figure 6:**
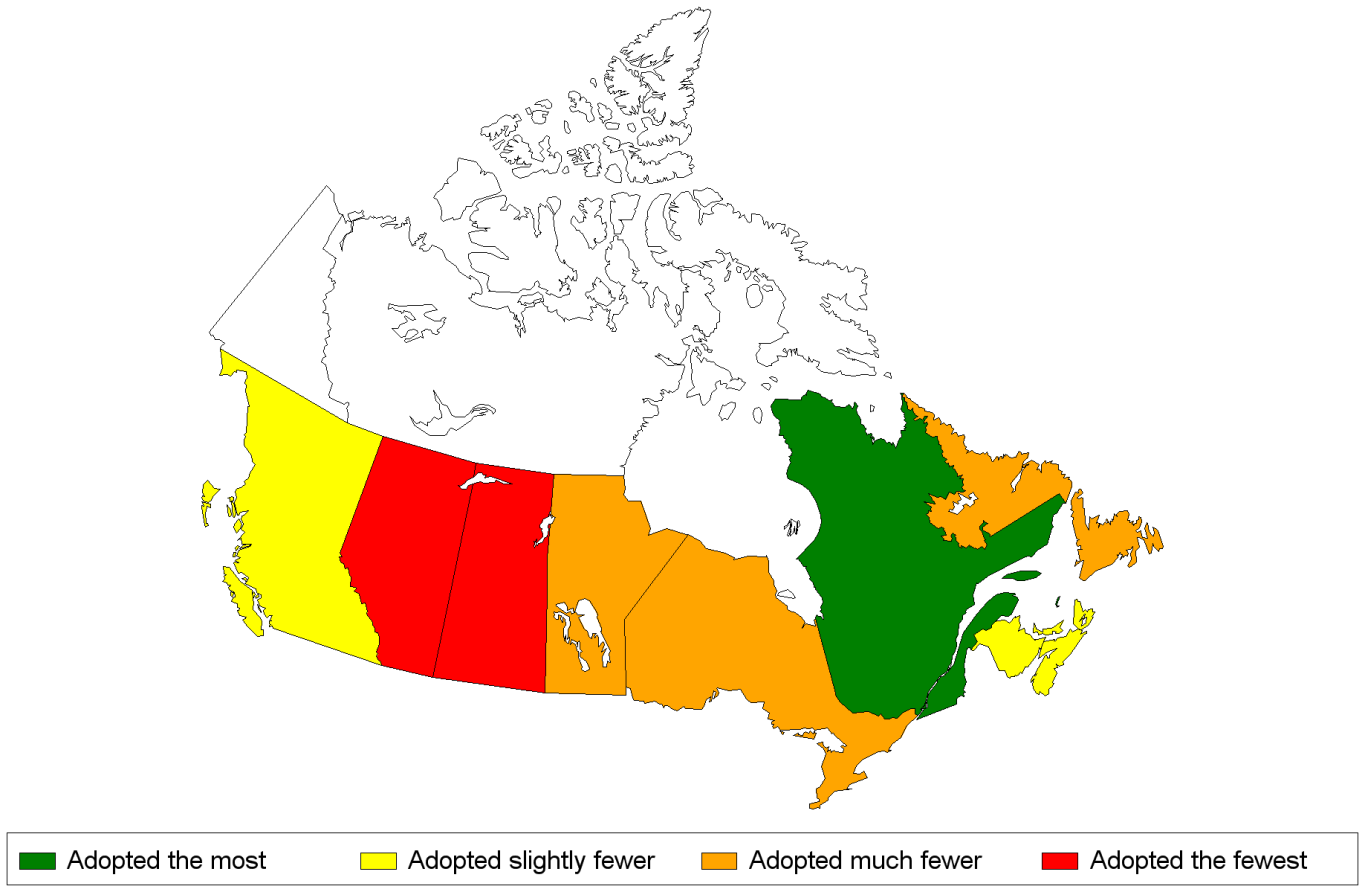
Provinces' classification according to the four categories of pro-environmental behaviours, Outdoor Index, 2007. **Legend**: from left to right – British-Columbia, Alberta, Saskatchewan, Manitoba, Ontario, Quebec, New-Brunswick, Nova-Scotia. Prince-Edward-Island is North of the two latter provinces and Newfoundland-and-Labrador is located North-East of Quebec.

The cluster analysis and dendogram plot resulted in the classification of each province into one of four categories reflecting different levels of adoption of pro-environmental behaviours: 1) adopting the most; 2) adopting slightly fewer; 3) adopting much fewer and; 4) adopting the fewest. The provincial coordinates and the categories generated from the cluster analysis are shown in Table 3 and Table 4. Maps of the Canadian provinces with their categories of pro-environmental behaviours are shown in Figure 5 and Figure 6.

**Table 3:** Provinces' coordinates on the Indoor Index, 2007.

Provinces	Coordinates	Categories^a^
Prince-Edward-Island	−0,0262	++
Nova-Scotia	−0,0179	++
Ontario	−0,0130	++
British-Columbia	−0,0055	+
Quebec	−0,0029	+
Alberta	0,0159	−
Manitoba	0,0225	−
Saskatchewan	0,0363	−
New-Brunswick	0,0381	−
Newfoundland-and-Labrador	0,0853	−

a Categories are: adopted the most pro-environmental behaviours (++), adopted slightly fewer (+), adopted much fewer (−) and adopted the fewest (−).

**Table 4:** Provinces' coordinates on the Outdoor Index, 2007.

Provinces	Coordinates	Categories^a^
Quebec	0,1038	++
Prince-Edward-Island	0,0261	+
Nova-Scotia	0,0123	+
New-Brunswick	0,0052	+
British-Columbia	0,0012	+
Manitoba	−0,0243	−
Newfoundland-and-Labrador	−0,0273	−
Ontario	−0,0350	−
Saskatchewan	−0,0887	−
Alberta	−0,0962	−

a Categories are: adopted the most pro-environmental behaviours (++), adopted slightly fewer (+), adopted much fewer (−) and adopted the fewest (−).

None of the 10 provinces were classified in both indices as adopting the most pro-environmental behaviours. For the Indoor Index, Ontario (ON), Prince Edward Island (PEI) and Nova Scotia (NS) rated in the top category, British Columbia (BC) and Québec (QC) in the next, the three Prairie provinces and New Brunswick (NB) in the third and Newfoundland and Labrador (NL) in “adopting the fewest” category (Figure 5). For the Outdoor Index, QC scored in the top category with BC, NS, NB and PEI following in second, and Manitoba (MN), ON and NL in third, followed by Alberta (AB) and Saskatchewan (SK) in the bottom category (Figure 6).

Four provinces (BC, QC, NS and PEI) were classified in the top two categories for both indices while four provinces (AB, SK, MN and NL) were classified for both indices, in the two lower categories.

### Composite indices by socio-demographic variables

The coordinates and the classification for the six comparison variables are shown in Table 5. For household income, educational attainment and number of persons in the household, there were oppositions in the classification of the responses. Households with a higher income, or higher educational attainment, or greater number of persons adopted more indoor pro-environmental behaviours, while those with a lower household income, educational attainment, or number of people, adopted more outdoor such behaviours. As well, households with water meters tended to adopt more indoor pro-environmental behaviours than those without, but for outdoors behaviours, the opposite applied – not having a water meter was associated with better adoption of pro-environmental behaviours. And finally, the dwelling's year of construction did not influence the adoption of pro-environmental behaviours as there was no trend on either index (Table 5).

**Table 5:** Coordinates and categories of pro-environmental behaviours for other socio-demographic variables, Indoor and Outdoor Indices, 2007.

	Indoor Index		Outdoor Index	
	Coordinates	Categories^a^	Coordinates	Categories^a^
**Household income**				
Less than $40,000	0,0253	−	0,1533	++
$40,000 to less than $80,000	−0,0080	+	−0,0133	−
$80,000 and over	−0,0273	++	−0,1567	−
**Highest education level**				
Secondary diploma or less	0,0228	−	0,0927	++
Postsecondary certificate or diploma	−0,0030	+	−0,0134	−
University	−0,0159	++	−0,0451	−
**Dwelling type**				
Apartment	0,0370	−	0,4335	++
Others	−0,0144	++	−0,1501	−
**Number of persons in the dwelling**				
One	0,0260	−	0,2058	++
Two	−0,0073	+	−0,0255	−
Three	−0,0104	++	−0,0723	−
Four or more	−0,0163	++	−0,1383	−
**Water meter**				
Yes	−0,0201	++	−0,1682	−
No	0,0123	−	0,1497	++
**Year the dwelling was built**				
Before 1946	−0,0089	+	0,0034	+
Between 1946 and 1960	−0,0040	+	−0,0130	−
Between 1961 and 1977	−0,0009	+	0,0025	+
Between 1978 and 1983	−0,0054	+	−0,0218	−
Between 1984 and 1995	−0,0084	+	−0,0364	−
Between 1996 and 2000	−0,0088	+	−0,0434	−
Between 2001 and 2005	−0,0099	++	−0,0948	−
2006 or latter	0,0042	+	0,0384	+

a Categories are: adopted the most pro-environmental behaviours (++), adopted slightly fewer (+), adopted much fewer (−) and adopted the fewest (−).

## Discussion

This study sought to develop a composite index which measures the overall adoption of pro-environmental behaviours among Canadian households. MCA, our main analytical technique, was used to aggregate survey data and to provide weights to the responses in the construction of the index. Our approach is similar to other studies in different fields [19]–[23]. This was followed by a cluster analysis to classify the provinces, as well as an exploration of relationships with socio-demographic factors.

The MCA generated two indices based on 39 of the 55 behavioural questions, an Indoor Index and an Outdoor Index, each reflecting environmental behaviours for 5 of the 12 survey themes. Retaining both indices allowed for better representation of the survey; together they cover 9 themes out of 12 (water use is in both) whereas one single index would have covered only 5, excluding important environmental themes such as fertilizer and pesticide use. As well, because the provincial classifications were different for each index and varied as well in the classification by socio-demographics factors for each index (e.g., household income) it was deemed justifiable to keep both indices.

Most (19/23) questions included in the Indoor Index were asked to all households with the exception of questions on recycling where only those households with access to a program were asked to respond. For the Outdoor Index, most questions (11/16) concerned watering of the lawn or the garden, and the use of fertilizers or pesticides. These (11) questions were answered only by households having a yard. However, even if households living in an apartment did not have to answer these questions, they were still recorded in the Index as households adopting pro-environmental behaviours (i.e., most valid skips were classified as pro-environmental responses and some as anti-environmental ones).

### The Indoor Index

One likely explanation for PEI and NS being classified in the top category for the Indoor Index is that nearly 100% of their households recycle and the proportion that compost is substantially above the Canadian average, as reported by Statistics Canada. In these two provinces, households are obligated by law to recycle and compost [25]. Moreover, questions regarding recycling contributed the most to the Indoor Index.

Recycling and composting are also common in ON but its good ranking is also related to the proportion of households that adopt water conservation behaviours (i.e. use water-efficient shower heads and toilets, run dishwasher and washing machine only when full) [7]. Provinces have been slowly adopting a provincial plumbing code requiring that new buildings use water-saving fixtures, with the exception of NL [26]; [27]. ON however was the first to adopt such a code in 1996 [26]; [28], and saw an increase in new residential construction from 1996 to 2002 [29], likely contributing to the higher proportion of households practicing water conservation behaviours [28]. This is an example where building codes may be effective in beneficially influencing the passive uptake of pro-environmental practices.

QC's good classification in the Indoor Index is in part due to its proportion of households adopting recycling behaviours being higher than the Canadian average. There were four questions on recycling which contributed significantly to the first dimension, thus contributing to QC's classification. Despite QC having the lowest proportion of households that compost [9] or participate in alternative recycling activities such as donations of furniture and clothing, QC's classification was only slightly affected as these behaviours had only moderate or low contributions to the Index.

In AB, MN, SK and NB, the proportion of households that adopted indoor pro-environmental behaviours is below the Canadian average (data not shown), explaining their lower classification in the Indoor Index. NL had only a few variables above the Canadian average and had most often the lowest proportion of all provinces. For example, the proportion is below the average for all four questions on water conservation and for all questions on recycling. In this province, there is no provincial plumbing code requiring the use of water-saving fixtures in new buildings [26]; [27]. Also, the proportion of households with access to a recycling program is only 71% [25].

### The Outdoor Index

Results for the Outdoor Index show a pattern with respect to Coastal proximity, with coastal provinces, with the exception of NL, rating in the two higher categories, and the continental provinces in the two lower categories with the two lowest rated provinces situated in the Prairies. The climate of the Prairies grasslands is characterized by hot summers combined with low precipitation and periodic drought. The climatic region of the Maritimes however is the one with the greatest annual precipitation [30]-[32], a pattern which is likely reflected in the frequency of watering lawn and or garden. Although watering of the lawn or garden is around the Canadian average in NL, its inhabitants own more recreational vehicles, use more gas and burn more yard waste on the property (data not shown) which may explain its lower classification than the other coastal provinces.

Also, there was an important difference in the proportion of households that used fertilizers and pesticides and QC had, by far, the lowest proportion. QC was the first province to adopt a provincial law in 2006 prohibiting the sale of pesticides for cosmetic purposes [7]; [33]. The Prairies on the other hand had the highest proportions of households that used pesticides or fertilizers in 2007 [7]; [10]. Subsequently, other jurisdictions have adopted similar laws begging the question of whether their classifications in the Outdoor Index will change over time.

It should also be noted that QC and BC have the highest proportion of households living in an apartment [10]. Given that most households living in an apartment do not have a backyard, they do not water neither lawn nor garden, nor do they use pesticides outdoors. Hence, they passively adopt pro-environmental behaviours and are considered as such by the MCA. In fact, these responses, recorded as ‘valid skip’, had the highest contribution to the Index, likely contributing to the higher classification for BC and QC on the Outdoor Index. Such passive behaviours or external factors were not excluded from the Index as they significantly contribute to the preservation of environmental resources.

### Indices for socio-demographic variables

For most socio-demographic variables, there were oppositions in the classification of the modalities, which means that it is not the same households that adopt pro-environmental behaviours on both indices. Higher income households may be more able to maintain and repair their housing and also invest in environmentally friendly products such as water and energy efficient appliances or fixtures, which can be more expensive than their regular counterparts [34]; [35]. Access to such products may contribute to the better classification on the Indoor Index for higher income households. On the other hand, lower income households may be less willing to pay water taxes linked to consumption levels, or to buy chemical products for their lawn or garden. Furthermore, those lower income households live more frequently in apartments where they do not have a yard, and they also own fewer recreational vehicles (data not shown). All these factors likely weigh in on the higher classification attributed to lower versus higher income households on the Outdoor Index.

In Canada, income is usually positively associated to educational attainment [36]. Also, the number of persons in a household will influence the household income. In the HES database, there was a significant correlation between households' income and education level as well as one with the households' income and the number of persons in the households (data not shown). This may explain why the indices by educational level and by number of persons in a household are similar to the one by household income. Any one of these three socio-demographic variables could potentially be used as a surrogate for the other two for future data collection for following Index trends over time.

Studies have shown that water meters with appropriate pricing are an incentive to reduce water consumption [2]. The US EPA estimated a 20% reduction in water consumption with universal metering [37] and a Canadian study also estimated a similar reduction according to structured water pricing [38]. While our results showed that households with water meters tended to score higher on the Indoor Index, households without a water meter scored higher on the Outdoor Index, which is in contrast to the other studies. We estimated that only 9% of households living in an apartment have water meters as opposed to 58% for all other types of dwellings in Canada (data not shown). As stated earlier, a household living in an apartment passively adopts more outdoor pro-environmental behaviours for lack of a lawn or garden to maintain with only a few having a water meter, possibly explaining the discrepancy between our results and those of other studies.

### Factors that can lead to pro-environmental behaviours

There is a wide variety of measures or instruments than can be introduced by governments to influence households behaviours, from economic instruments to direct regulation, labeling, information campaigns and provision of environment-friendly public goods such as public transportation or bicycle paths [39].

This study has identified factors which seem to influence the uptake of beneficial environmental behaviours at the household level. Investment in infrastructure is one of them. The physical or material possibility to act pro-environmentally must indeed be available [40], such as what might be needed for Newfoundlanders to improve their recycling profile.

Regulation is frequently used to efficiently influence the environmental impacts of household decision-making [39] and in our study it also seems to be an important incentive for the adoption of pro-environmental behaviours. This was seen both in the case of building codes requiring the installation of water efficient shower heads and toilets, and in the case of the ban on pesticides for lawn care. In Ontario, the ban of cosmetic pesticides decreased significantly the concentration of some pesticides, mainly herbicides, in the majority of streams under surveillance near urban areas with limited agriculture activities [41].

To encourage a reduction in water consumption, both price and non-price policies should be used. Volumetric water charges are associated with both water-saving behaviours and adoption of water-efficient devices [39]. However, in a study in several OECD countries, Canada had the highest proportion of households that did not know how they were charged for residential water consumption, thus reducing the price effect on water-saving behaviours [39]. In our study, presence of water meters was an incentive to water-saving behaviours only for indoor behaviours. Climate was also another factor that could be influential. Hence, public information on the environmental impact of water consumption and on measures households can adopt to save water should be combined to economic measures according to the OECD [39] and this study.

Other than governmental measures, household characteristics may play a role in the adoption of environment-related behaviours such as income, household composition and dwelling characteristics [39]. According to the OECD survey, low income households and tenants households make fewer financial investments in water efficiency, as can be expected. Grants targeted at those households to correct the economic imbalance are thus recommended by the agency. Moreover, our study showed households from both income groups (high or low) or dwelling type (owned or rented) have to improve their act in different domains and that programs should target them accordingly. In short, Canadians remain very dependent for many such actions on where they live and what the climate brings to their yards, or not.

### Limits of the study

We used data from a survey that has been created to address the needs of Statistics Canada and the federal government. Thus, we were limited to its content. The questionnaire does not cover all behaviours that can impact the environment and public health. Also, the indices developed here measure the behaviours available in the survey and retained after the analysis by an expert group for their potential positive impacts on health, and not all existing pro-environmental behaviours. The classification could have been different if other behaviours had been included.

Three themes of the survey were not covered by the indices, namely dwelling characteristics, motor vehicle and indoor environment. However, we believe they would not have much impact in the indices. First, there were no behaviours measured in the dwelling characteristics theme and some of the characteristics were included as passive variables in the indices (e.g. year the building was built). The same happened for the motor vehicle theme (focused on the characteristic of the car), yet we used another theme to include the number of vehicles owned by the households in the outdoor index. For the indoor environment theme, only 2 of the 5 questions measured behaviours and they both concerned the type of chemical products used to clean windows and the dwelling. Although every small action is important for the environment, those questions were excluded as some other practices, such as agriculture, use similar products in much larger quantities [42].

A good standing in the classification does not mean that there is no place for improvement. Indeed, a high proportion of households that adopt pro-environmental behaviours on one question can compensate for a lower proportion on another question of the same index. Also, the provinces were compared to each other and not classified in relation to a gold standard.

Furthermore, it was the MCA that attributed the weight for each modality. Thus, a modality with a higher weight has more impact in the index. For instance, all four recycling questions had the highest contribution to the indoor index. Further studies should investigate if the inclusion of only one of those recycling question or a composite index of those four questions would be more appropriate. The same reasoning should also be applied to questions related to the watering of the lawn or of the garden. Households without a garden or a lawn are rewarded for every question on that subject which at the end can impact greatly the province classification. For example, they were not only rewarded for not watering their lawn, but they were also rewarded for the question concerning the watering duration and the number of time they water. Because the MCA attributed the weights, those household without a garden or lawn had a higher ‘reward’ that households with a garden or lawn that did not water them.

One general limit of MCA is that the first dimension usually explains a low proportion of the total inertia in the data set and the other dimensions explain less than the first [18]. In this study, the first dimension explained 33% and 62% of the total inertia for the indoor and outdoor index respectively. By using only the first dimension, these indices might not properly reflect all of the behaviours, especially for the indoor one. However, using more than one dimension to build the index would not considerably increase the total inertia explained but would in return increase its complexity. Composite indices are indeed built to simplify the analysis.

Because of the study design, based on households, it was also not possible to evaluate the impact of personal attributes, like age and gender, on the adoption of environmental behaviours. The association between environmental behaviours and age is not clear. Studies have observed all possible trends, from older people adopting more pro-environmental behaviours to the opposite trends or no trend at all [43]–[45]. Also, women would be more likely to take pro-environmental actions than men, although some studies have found the opposite depending on behaviour and region [43]–[46]. In our study we found that socio-demographic characteristics like household income and a higher level of education did not have the same influence on outdoor behaviours compared to indoor behaviours. Hence, some differences could also be expected between indoor and outdoor behaviours for age and gender.

Because the survey was not meant to measure attitudes or values, we cannot associate the classification of the province to any difference in values or perception. However, others studies have showed cultural differences across Canadian provinces [47]–[51]. In Canada, French speaking people are at majority in the province of Quebec but a minority in the rest of Canada as opposed to English speaking Canadian that are a majority in the rest of Canada [52]; [53]. Several studies have observed differences of values and attitudes in terms of personality, political perspective, priorities and social issues between English-Canadians and French-Canadians [47]–[51]. Differences in those values could also explain some differences in the adoption of pro-environmental behaviours but further studies are required to confirm it.

Attitudes and values can also be different in immigrants compared to the native born. The former usually have a smaller ecological footprint [54]–[57]. For example, several studies, mostly from United States, have observed that immigrants have lifestyles that are less demanding on the environment: they consume less, possess fewer luxury items like SUVs, they carpool or use public transportation more often and live in smaller houses [54]–[57]. In 2006, around 55% of all Canadian immigrants were in Ontario, followed by 18% in British-Columbia and 14% in Quebec [58]. British-Columbia and Quebec had a good classification on both indices. However, we could not estimate the impact of immigration on these classifications, as immigration rules and influx have changed significantly over the last decades [58].

Despite those limits, the indices still give a good idea of the global adoption of pro-environmental behaviours with potential positive impacts on health in Canada and remain easy to explain and understand. The main sectors in which households can have an impact are covered by the indices, like air and soil quality as well as water conservation. The weighting methods used (i.e. MCA) are also more appropriate to assign weights as opposed to an equal weights or expert opinion approach that are often criticized for being arbitrary or simplistic [22]. Others similar indices could be created as the survey is performed every two years. The results obtained with the 2007 indices could serve as the baseline for surveillance purposes, as the survey has been more comprehensive since that date.

## Conclusion

MCA was successfully applied in creating Indoor and Outdoor composite Indices of environmental health relevance based on a readily available periodic Statistics Canada dataset. The Indices cover a good range of environmental themes at the household level and the analysis, particularly the indices weights obtained in the MCA, could be applied to similar surveys worldwide (as baseline weights) enabling temporal trend comparisons for recurring themes. Results uncovered provincial patterns of pro-environmental behaviours adoption with certain provinces scoring consistently higher and others consistently lower, as well as the associations between socio-demographic factors and the indices. Much more than voluntary measures, this study shows that existing regulations, dwelling type, household composition and income as well as climate are the major factors determining pro-environmental behaviours.

## Supporting Information

Figure S1 **Map representation of the MCA results on the 55 questions with extreme responses.**
(TIFF)

Table S1 Results of the MCA on the 55 questions without extreme responses (exploratory analysis). **Legend:** N/A: Results are not available for supplementary variables. Qlt: Quality (i.e. the sum of the squared correlations for the first two dimensions in this case). Inr: Inertias. K: Principal coordinates for the first dimension. Cor: Squared correlation with the first dimension. Ctr: Contributions of the modality to the explained inertia of the first dimension. All cells are multiplied by 1000. Results are the same on rows and on column when a Burt table is used.
(XLS)

Table S2 Results of the MCA for the Indoor Index, 2007. **Legend:** N/A: Results are not available for supplementary variables. Qlt: Quality (i.e. the sum of the squared correlations for the first two dimensions in this case). Inr: Inertias. K: Principal coordinates for the first dimension. Cor: Squared correlation with the first dimension. Ctr: Contributions of the modality to the explained inertia of the first dimension. All cells are multiplied by 1000. Results are the same on rows and on column when a Burt table is used.
(XLS)

Table S3 Results of the MCA for the Outdoor Index, 2007. **Legend:** N/A: Results are not available for supplementary variables. Qlt: Quality (i.e. the sum of the squared correlations for the first two dimensions in this case). Inr: Inertias. K: Principal coordinates for the first dimension. Cor: Squared correlation with the first dimension. Ctr: Contributions of the modality to the explained inertia of the first dimension. All cells are multiplied by 1000. Results are the same on rows and on column when a Burt table is used.
(XLS)

Table S4 Questions and responses selected for the Indoor Index, 2007.
(DOC)

Table S5 Questions and responses selected for the Outdoor Index, 2007.
(DOC)
