# Supplementary figures and images for: Development of Composite Indices to Measure the Adoption of Pro-Environmental Behaviours across Canadian Provinces
Source: PLoS One. 2014 Jul 11;9(7):e101569. doi: 10.1371/journal.pone.0101569 (PMC4094473; doi:10.1371/journal.pone.0101569)

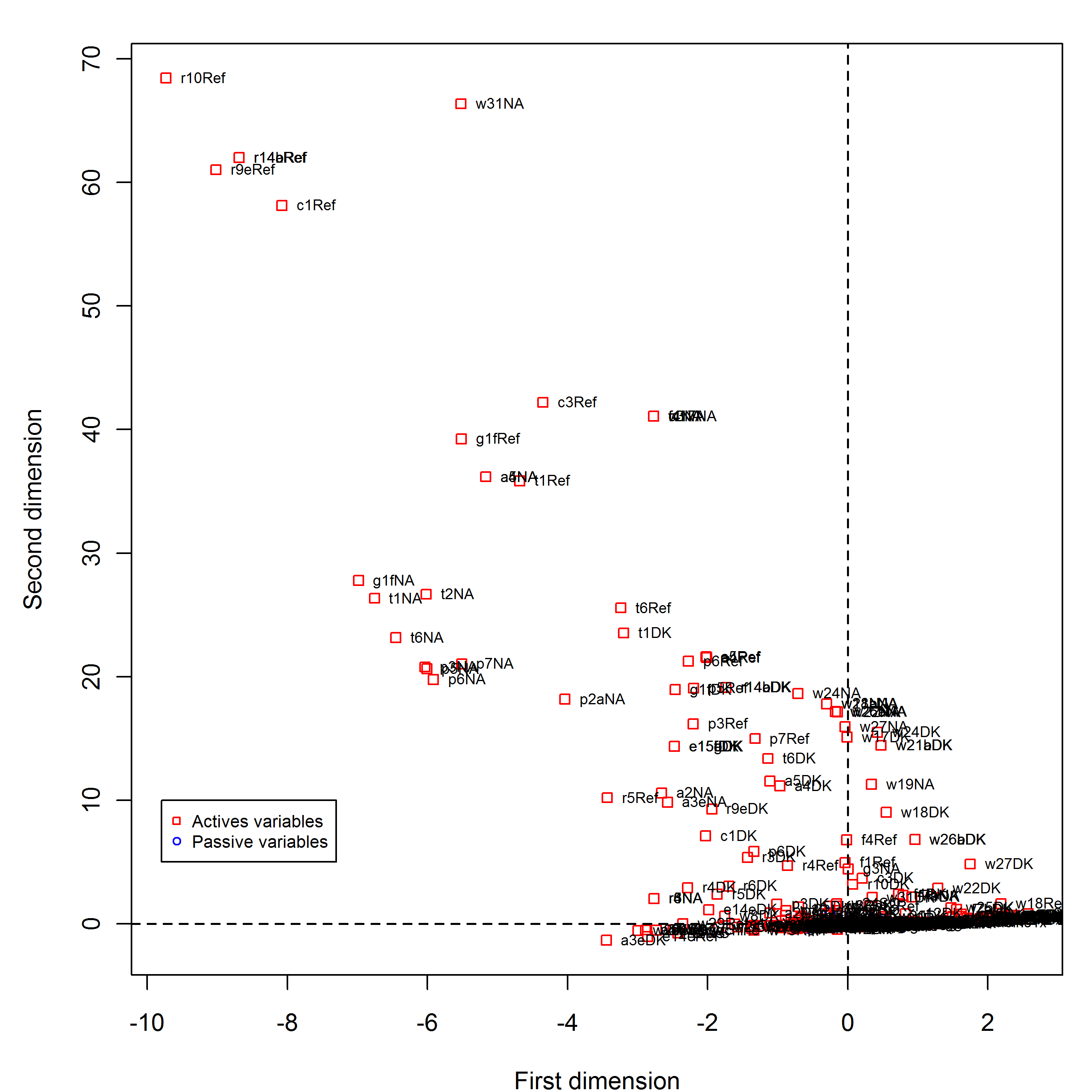

Supplement: Figure S1 — Map representation of the MCA results on the 55 questions with extreme responses. (TIFF) [file pone.0101569.s001.tiff]
