# Supplementary material for: Development of Composite Indices to Measure the Adoption of Pro-Environmental Behaviours across Canadian Provinces
Source: PLoS One. 2014 Jul 11;9(7):e101569. doi: 10.1371/journal.pone.0101569 (PMC4094473; doi:10.1371/journal.pone.0101569)
Supplement: Table S4 — Questions and responses selected for the Indoor Index, 2007. (DOC) [file pone.0101569.s005.doc]

**Table S4. Questions and responses selected for the Indoor Index, 2007.**

| **Questions^a^** | **Responses selected^b^** | | **Responses excluded** |
| --- | --- | --- | --- |
|  | **Pro-env.** | **Anti-env.** |  |
| EH_Q14e Does not use at least one of these energy saving lights: Compact fluorescent lights, florescent tubes, halogen lights, LED holiday lights | No | Yes | Don’t know, refusal |
| EH_Q15a Use dimmers on households lights | Yes | No | Don’t know |
| EH_15b Unplug electronics when away for an extended period of time | Yes | No | Don’t know |
| EH_15c Reduce heating or cooling in certain areas of the dwelling | Yes | No | Don’t know |
| EH_15d Use a clothesline or drying rack to dry clothing | Yes | No | Don’t know |
| EH_15f Close the blinds or drapes in the dwelling during the hottest part of the day | Yes | No | Don’t know |
| EH_15g Put plastic film on the windows in the winter | Yes | No | Don’t know |
| EH_15h Put on more clothing, such as sweater, instead of adjusting the temperature | Yes | No | Don’t know |
| WA_Q14 Use a water saving low flow showerhead | Yes | No | Don’t know, refusal |
| WA_Q15 Use a low volume toilet or a toilet tank with the water volume modified | Yes | No | Don’t know, refusal |
| WA_Q29 Frequency that the washing machine was full before it was turned on, in the last 12 months | Always or often, no machine | Sometimes or rarely or never | Don’t know, refusal |
| WA_Q30 Frequency that the dishwasher was full before it was turned on, in the last 12 months | Always or often, no dishwasher | Sometimes or rarely or never | Don’t know, refusal |
| RC_Q03 Quantity of recyclable paper waste that was recycle, in a average week in the last 12 months^c^ | All or most | Some or none, valid skip | Don’t know, refusal, not stated |

| **Questions^a^** | **Responses selected^b^** | | **Responses excluded** |
| --- | --- | --- | --- |
|  | **Pro-env.** | **Anti-env.** |  |
| RC_Q04 Quantity of recyclable plastic waste that was recycle, in a average week in the last 12 months^c^ | All or most | Some or none, valid skip | Don’t know, refusal, not stated |
| RC_Q05 Quantity of recyclable glass waste that was recycle, in a average week in the last 12 months^c^ | All or most | Some or none, valid skip | Don’t know, refusal, not stated |
| RC_Q06 Quantity of recyclable metal waste that was recycle, in a average week in the last 12 months^c^ | All or most | Some or none, valid skip | Don’t know, refusal, not stated |
| RC_Q09e Return none of the following materials for refund, in the last 12 months: beer, liquor or wine bottles, soft drink or beer cans, other glass or plastic bottles | No | Yes | Don’t know, refusal |
| RC_Q10 Took any products such as unwanted or used electronics, paint, oil, antifreeze, expired medication or batteries back to a store or supplier | Yes | No | Don’t know, refusal |
| RC_Q14a Participated in a alternative recycling activity in the last 12 months: Donated or gave away used clothing | Yes | No | Don’t know, refusal |
| RC_Q14b Participated in a alternative recycling activity in the last 12 months: Donated or gave away used furniture or appliances | Yes | No | Don’t know, refusal |
| RC_Q14c Participated in a alternative recycling activity in the last 12 months: Donated or gave away used electronic’s such as TV’s or computers | Yes | No | Don’t know, refusal |

| **Questions^a^** | **Responses selected^b^** | | **Responses excluded** |
| --- | --- | --- | --- |
|  | **Pro-env.** | **Anti-env.** |  |
| CP_Q01 Separate any kitchen waste from the rest of the garbage and put it out for compost collection, take it to a depot or put it in a compost bin or pile, in the past 12 months | Yes | No | Don’t know, refusal |
| PD_Q03 Frequency for which the households purchased brand name items that was perceived to be better quality, even though they may be more expensive than other similar products | Always or often | Sometimes or rarely or never | Don’t know, refusal, not stated |

^a^ In this table, questions were synthesize compared to the official 2007 HES questionnaire.

^b^ Responses used in the final multiple correspondence analysis.

c The question is answer only by households that have access to a recycling program.
