# Supplementary material for: Development of Composite Indices to Measure the Adoption of Pro-Environmental Behaviours across Canadian Provinces
Source: PLoS One. 2014 Jul 11;9(7):e101569. doi: 10.1371/journal.pone.0101569 (PMC4094473; doi:10.1371/journal.pone.0101569)
Supplement: Table S5 — Questions and responses selected for the Outdoor Index, 2007. (DOC) [file pone.0101569.s006.doc]

**Table S5. Questions and responses selected for the Outdoor Index, 2007.**

| **Questions^a^** | **Responses selected^b^** | | **Responses excluded** |
| --- | --- | --- | --- |
|  | **Pro-env.** | **Anti-env.** |  |
| AQ_Q04 Burn yard waste on the property, in the last 12 months^c^ | No, valid skip | Yes | Don’t know, refusal, not stated |
| GP_Q01F Does not own any of the following recreational vehicles, in the last 12 months: All-terrain vehicle, snowmobile, dirt bike or motocross motorcycle, personal watercraft, motorboat | Yes | No | Don’t know, refusal, not stated |
| GP_Q03 Approximate fuel quantity used in operating recreational vehicles, in the last 12 months^d^ | Less than 101 litres, valid skip | 101 litres or more | Not stated |
| TD_Q01 Own or lease a motor vehicle for personal use, in the past 12 months | No | Yes | Don’t know, refusal, not stated |
| TD_Q02 Number of vehicle own or lease for personal use, in the last 12 months^e^ | 1 or 2 vehicles, valid skip | 3 or more vehicles | Don’t know, refusal, not stated |
| TD_Q06 Use regularly (1 to 3 times per week) public transport transit other that for travel to work, in the last 12 months^f^ | Yes | No | Don’t know, refusal, not stated |
| WA_Q17 Water the lawn last summer^g^ | No, valid skip | Yes | Don’t know, not stated |
| WA_Q18 Number of time the lawn was watered in an average week^h^ | Once a week or less, valid skip | Twice or more a week | Don’t know, refusal, not stated |
| WA_Q19 Average duration of a watering session^h^ | Less than 30 minutes, valid skip | 30 minutes or more | Don’t know, not stated |
| WA_Q21a Water the lawn by hand using a watering can or a hose^h^ | Yes, valid skip | No | Don’t know, not stated |
| WA_Q24 Water the garden or areas with threes, shrubs, flowers or vegetables last summer^i^ | No, valid skip | Yes | Don’t know, not stated |

| **Questions^a^** | **Responses selected^b^** | | **Responses excluded** |
| --- | --- | --- | --- |
|  | **Pro-env.** | **Anti-env.** |  |
| WA_Q25 Number of time these areas were watered in an average week^j^ | Once a week or less, valid skip | Twice or more a week | Don’t know, refusal, not stated |
| WA_Q26a Water those areas by hand using a watering can or a hosej | Yes, valid skip | No | Don’t know, not stated |
| FP_Q01 Applied chemical fertilizers to the lawn or the garden, in the last 12 monthsk | No, valid skip | Yes | Don’t know, refusal, not stated |
| FP_Q04 Applied chemical pesticides to the lawn or the garden, in the last 12 monthsk | No, valid skip | Yes | Don’t know, refusal, not stated |
| FP_Q07a Applied pesticides as part of a regular maintenance schedulel | No, valid skip | Yes | Don’t know, refusal, not stated |

^a^ In this table, questions were synthesize compared to the official 2007 HES questionnaire.

^b^ Responses used in the final multiple correspondence analysis.

The question is answer only by respondent or households:

^c^ who do not live in an apartment.

^d^ who owned recreational vehicles in the last 12 months.

^e^ who own or leased a motor vehicle in the past 12 months.

^f^ who have access to public transit.

^g^ who do not live in an apartment and who had a lawn last summer.

^h^ who do not live in an apartment and who had a lawn last summer and reported it as watered.

^i^ who do not live in an apartment who have a garden.

^j^ who do not live in an apartment that had a garden last summer and reported it as watered.

^k^ who do not live in an apartment and had a lawn or a garden last summer.

^l^  who do not live in an apartment and had a lawn or a garden last summer and had pesticides applied.
